# Developing a social vulnerability index for antenatal care screening of risk for adverse maternal and perinatal outcomes

**DOI:** 10.1002/ijgo.71138

**Published:** 2026-06-20

**Authors:** Gabriel Q. Rippel, Renato T. Souza, Jussara Mayrink, Maria J. Miele, Rafael B. Galvao, Maria L. Costa, Francisco E. Feitosa, Edilberto A. Rocha Filho, Debora F. Leite, Ricardo P. Tedesco, Danielly S. Santana, Karayna G. Fernandes, Janete Vettorazzi, Iracema M. Calderon, Jose G. Cecatti

**Affiliations:** ^1^ Department of Obstetrics and Gynaecology, School of Medical Sciences University of Campinas Campinas São Paulo Brazil; ^2^ Department of Gynecology and Obstetrics Federal University of Ceará Fortaleza Ceará Brazil; ^3^ Department of Gynecology and Obstetrics, Medical Sciences School Federal University of Pernambuco Recife Pernambuco Brazil; ^4^ Department of Obstetrics and Gynecology Jundiaí Medical School Jundiaí São Paulo Brazil; ^5^ Department of Obstetrics and Gynecology, Maternity of the Clinic Hospital Federal University of RS Porto Alegre Brazil; ^6^ Department of Gynecology and Obstetrics Botucatu Medical School (UNESP) Botucatu Brazil

**Keywords:** antenatal care, maternal outcomes, perinatal outcomes, pregnancy, social vulnerability

## Abstract

**Objective:**

Social determinants of health influence maternal and perinatal outcomes, yet tools to operationalize these risks in clinical care remain scarce. We aimed to study social determinants in Brazilian pregnant women and develop a social vulnerability index (SVI) that could correlate with pregnancy and perinatal outcomes.

**Methods:**

The present study was a secondary analysis of 1565 low‐risk nulliparous women enrolled in two Brazilian cohort studies. We selected vulnerability indicators from sociodemographic data and tested the performance and risk association of multiple SVI models with any adverse outcome (preterm birth, gestational diabetes mellitus, pre‐eclampsia, small or large for gestational age, low 5‐min Apgar score, neonatal intubation, neonatal intensive care unit admission, fetal or neonatal death) using chi‐square tests, logistic regression, and receiver operating characteristic analysis.

**Results:**

Advanced maternal age, non‐white ethnicity, and exclusive publicly funded antenatal care were the most consistent vulnerability predictors of adverse outcomes. The final three‐variable SVI demonstrated a significant dose–response gradient, with maternal adverse outcomes increasing from 16.4% (no vulnerabilities) to 43.8% (3 vulnerabilities) and perinatal adverse outcomes rising from 22.1% to 35.6%. The model presented a sensitivity of 64.71%, a specificity of 42.56%, a positive predictive value of 47.46% and a negative predictive value of 60.07% for any adverse outcome.

**Conclusion:**

The three‐variable SVI offers a simple, reproducible, and context‐adapted screening tool for primary care. Either for individual or population screening, it can be easily combined with clinical risk assessment, targeting those who may benefit from equity‐oriented maternal health strategies.

## 1 INTRODUCTION

Since its foundation in 1948, the WHOs constitution has acknowledged the influence of social and political conditions on health, promoting actions to strengthen equity under the banner of “Health for All.” Building on this principle, the Commission on Social Determinants of Health was later established to translate emerging evidence into policy. In 2010, this process culminated with the publication of the Conceptual Framework for Action on the Social Determinants of Health, which identified three key domains of vulnerability: the socioeconomic and political context, structural determinants of socioeconomic position, and intermediary determinants.^1^ Moreover, the article described the impact of these determinants as a life‐long exposure that permeates the health of an individual and is exacerbated during moments of frailty.^1^

Social vulnerability (SV) refers to the accumulation of multiple risk factors arising from limited material or social resources that may be responsible for compromising people's well‐being.^2^ Evidence shows that higher levels of SV increase frailty, reduce resilience against illness, and are independently linked to adverse clinical outcomes and higher mortality rates.^3^, ^4^

These social determinants of health have a significant impact during pregnancy, and increasing evidence suggests that the parental and familial social context has a strong influence on both maternal outcomes and newborn health.^5^, ^6^, ^7^, ^8^, ^9^ However, despite a broad consensus on this association, quantifying its impact and determining how much each factor contributes to an individual patient's outcome remains a methodological challenge. In literature, there are multiple ways that authors use to approach this issue; some rely on deprivation indices based on national demographic data by neighborhood,^10^, ^11^, ^12^ others are based on retrospective individual patient data,^7^, ^8^ and some use a combination of both.^9^ In Brazil, some studies have developed social vulnerability indices (SVIs) based on population‐level data to guide public health policies,^13^, ^14^ but these approaches focus on demographic regions and are aimed at the general population, rather than on pregnant women specifically. Moreover, even the studies that aim to create a standardized SVI with predictive value often face additional challenges, mainly related to country‐specific conditions, which limit external reproducibility, and the difficulty in covering all protective and vulnerability factors and their specific weights.^6^

Although their importance to the health‐illness process among pregnant women is widely acknowledged,^15^ social vulnerabilities are shaped by multiple sociodemographic characteristics and diverse exposures, ranging from partner and family relations to financial constraints and women's self‐perception of their social role and clinical conditions.^16^

Given the complexity and heterogeneity of SV, especially in low‐ and middle‐income countries, we sought to explore its impact on maternal and perinatal health outcomes in a Brazilian population of pregnant women. Using data from two large multicenter cohorts, we aimed to define and stratify a SVI based on individual‐level factors to assess its correlation with adverse pregnancy and neonatal outcomes.

## 2 METHODS

This is an ancillary analysis joining data from two previously conducted Brazilian cohort studies—Preterm SAMB^17^ and MAES‐I^18^—with the specific objective of defining and stratifying a SVI tailored to Brazilian pregnant women. Using a total of 1565 cases of healthy pregnant nulliparous women included at mid‐pregnancy and followed until childbirth, we aim to develop an SVI based on self‐reported sociodemographic data and evaluate its distribution and association with adverse maternal and perinatal outcomes. After exclusion of incomplete records, 1553 cases were included in the maternal outcome analysis and 1490 in the perinatal outcome analysis (Figure 1).

**FIGURE 1:**
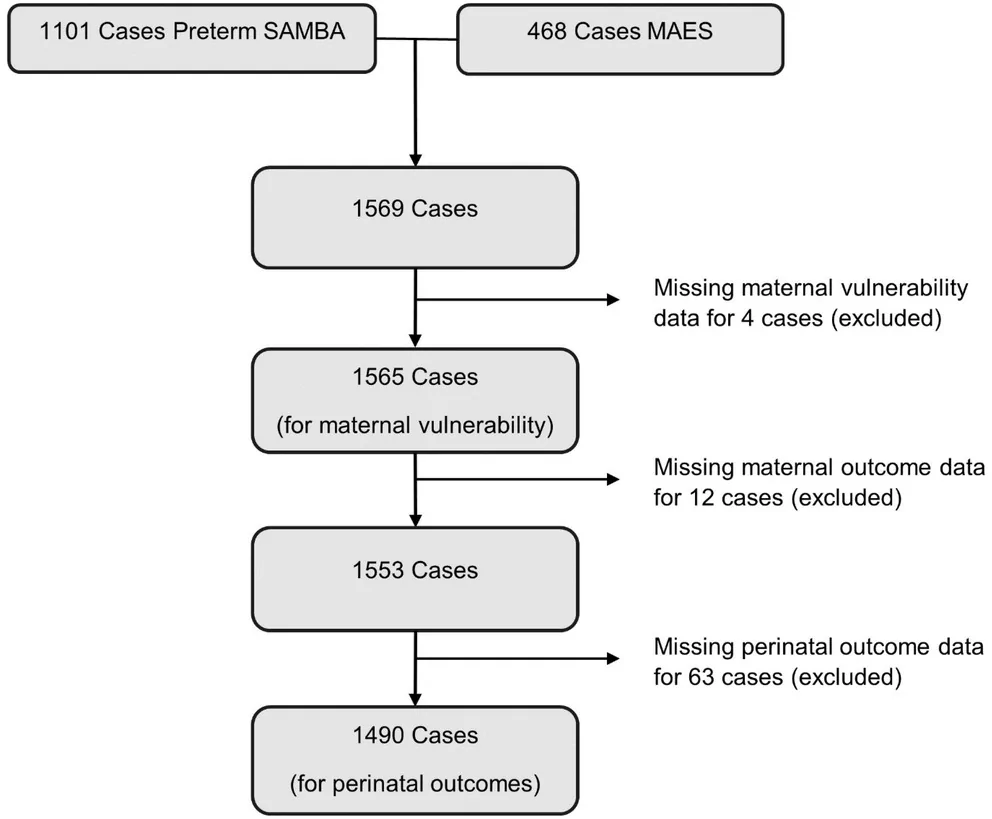
Flow chart of the population included in the study.

SV indicators were selected among available information from the original questionnaires and included maternal age (<20 or >34 years), ethnicity (non‐white), education (<12 years of schooling), low annual income (<6000 Brazilian real [BRL]/year), marital status (no partner), living status (alone), employment status (unemployed), drug use during pregnancy, and type of prenatal care (publicly funded only).

Maternal outcomes included preterm birth (PTB), gestational diabetes mellitus (GDM), pre‐eclampsia (PE) and the composite of any adverse maternal outcome (AMO), including all as described above. Perinatal outcomes encompassed birth weight classified as small or large for gestational age (SGA/LGA), low 5‐min Apgar score, neonatal intubation, neonatal intensive care unit (NICU) admission, fetal or neonatal death, and the composite any adverse perinatal outcome (APO), including all as described above. The definitions of these outcomes followed those used in the original cohort studies currently joined.^17^, ^18^

We initially evaluated the association between each SV indicator and AMO and perinatal outcomes using chi‐square tests and risk ratios (RR) with 95% confidence intervals (CIs). To assess potential collinearity and redundancy among variables and assess how they interact with each other, we performed multivariate logistic regression analysis. This exploratory process guided the selection of statistically relevant variables and weights for inclusion in different versions of the SVI.

We also created a variety of SVI test indices combining different vulnerabilities, including some that were not significant, to explore how various combinations affected correlations with outcomes. Each SVI test thus generated was then evaluated for correlation and predictive value of AMO and APO outcomes through chi‐square testing and receiver operating characteristic (ROC) curve analyses.

At this stage, two candidate SVI models showed the best predictive performance with very similar indicators: one including three variables (advanced maternal age, ethnicity, and type of prenatal care) and another including these plus family annual income. We then evaluated the performance of both SVI options in two steps. First, without weighted variables, by evaluating the absolute risk of AMO and APO for each value of the SVI test. Second, we performed ROC curve analyses for all three and four variable options to identify the model with the largest area under the receiver operating characteristic (AUROC) curve and the cutoff that provided optimal sensitivity and specificity for stratifying patients into high and low SVI groups. Finally, we evaluated the performance of the two best SVIs by measuring sensitivity, specificity, positive predictive value (PPV) and negative predictive value (NPV) for AMO, APO and any adverse outcome (AAO), that is, any AMO or APO.

All analyses were performed using SPSS version 21.0 (IBM Corp., Armonk, New York, USA). *P* values below 0.05 were considered statistically significant.

### 2.1 Ethical aspects

The Preterm SAMBA study has been reviewed and approved by the National Committee for Ethics in Research of Brazil (CONEP) and by the Institutional Review Board (IRB) of the coordinating center (letter of approval 1.048.565 issued on April 28, 2015), as well as by the IRBs of all other participating Brazilian centers. All women enrolled in the Preterm SAMBA Brazilian cohort signed a written informed consent form, which also allowed for future additional studies using their data and biological samples without requiring further consent. Following the same procedures, all women enrolled in the MAES‐I cohort signed an informed consent form. The ethical principles stated in the Brazilian National Health Council (Resolution CNS 466/12) were respected in every stage of this study. The anonymity of the source of information was guaranteed, and the care for the women was provided independent of their agreement to participate in the study. The methodological and ethical aspects of the Preterm SAMBA and MAES‐I study protocols were developed following STROBE guidelines.^19^

## 3 RESULTS

### 3.1 Bivariate analysis

The associations between individual SV indicators and AMO and perinatal outcomes are presented in Table 1. Findings are structured by outcome type and discussed in the context of relevant literature on maternal health and social determinants. Advanced maternal age (>34 years) was associated with increased risk for any AMO (RR 1.61; 95% CI: 1.18–2.20), while adolescent pregnancy (<20 years) showed a protective association (RR 0.76; 95% CI: 0.60–0.96). Women receiving exclusively funded public antenatal care had a borderline non‐significant increased risk of AMOs (RR 1.30; 95% CI: 0.95–1.77; *P* = 0.096).

**TABLE 1:** Distribution of AMO and APO according to individual social vulnerability indicators.

Sociodemographic characteristics		AMO (*n* = 1553)					APO (*n* = 1490)				
		Yes	No	RR	95% CI	*P* value	Yes	No	RR	95% CI	*P* value
Maternal age	<20 years	70	308	**0.761**	**0.602–0.964**	**0.020**	124	234	1.069	0.905–1.263	0.435
	20–34 years	269	837	Ref.			345	720	Ref.		
	>34 years	27	42	**1.609**	**1.178–2.198**	**0.006**	20	47	0.921	0.632–1.344	0.666
Marital status	Without partner	101	321	1.021	0.836–1.248	0.835	143	260	1.115	0.952–1.305	0.182
	With partner	265	866	Ref.			346	741	Ref.		
Living status	Alone	5	25	0.703	0.314–1.573	0.369	7	21	0.758	0.398–1.446	0.374
	Accompanied	361	1162	Ref.			482	980	Ref.		
Ethnic group	Non‐white	243	723	1.200	0.992–1.453	0.059	324	596	**1.217**	**1.041–1.421**	**0.012**
	White	123	464	Ref.			165	405	Ref.		
Years of study	<12 years	254	842	0.946	0.779–1.148	0.573	352	701	1.066	0.960–1.255	0.437
	≥12 years	112	345	Ref.			137	300	Ref.		
Annual income (<BRL 6000)	<BRL 6000	37	94	1.221	0.914–1.630	0.187	38	87	0.920	0.698–1.212	0.547
	≥BRL 6000	329	1093	Ref.			451	914	Ref.		
Annual income (<BRL 12 000)	<BRL 12 000	102	335	0.987	0.808–1.205	0.895	131	283	0.951	0.806–1.122	0.549
	≥BRL 12 000	264	852	Ref.			358	718	Ref.		
Labor activity	Not working	202	632	1.062	0.887–1.272	0.514	253	555	0.905	0.783–1.046	0.178
	Employed	164	555	Ref.			236	446	Ref.		
Type of antenatal care	Only public	331	1035	**1.295**	**0.947–1.771**	**0.096**	443	866	**1.332**	**1.026–1.728**	**0.024**
	Other	35	152	Ref.			46	135	Ref.		
Use of any drugs^a^	Current or during pregnancy	72	220	1.026	0.817–1.289	0.823	93	187	1.051	0.869–1.270	0.612
	Never	246	778	Ref.			312	675	Ref.		

*Note*: Values in bold are statistically significant.

Abbreviations: AMO, adverse maternal outcomes; APO, adverse perinatal outcomes; BRL, Brazilian real; CI, confidence interval; RR, risk ratio.

^a^ Missing information for 237 cases.

Regarding perinatal outcomes, non‐white ethnicity was significantly associated with increased risk of adverse events (RR 1.22; 95% CI: 1.04–1.42), as was public‐only antenatal care (RR 1.33; 95% CI: 1.03–1.73). Additional analyses showed that women ≥35 had a higher risk of developing GDM (RR 1.88; 95% CI: 1.12–3.16), while adolescents had a significantly lower risk (RR 0.45; 95% CI: 0.27–0.74) (data not shown in table). No additional significant associations were observed between other individual indicators (education, marital status, income, drug use) and adverse outcomes.

### 3.2 Multivariate analysis

Table 2 focuses on the findings of logistic regression calculations for vulnerability variables compared to AMO and APO. Advanced maternal age (>34 years) remained independently associated with a higher risk of AMOs (Exp[*B*] 2.48; 95% CI: 1.41–4.37). Contrarily, adolescent pregnancy showed a trend toward protection for maternal outcomes, though this association were no longer statistically significant after adjustment (Exp[*B*] 0.72; 95% CI: 0.50–1.02). Non‐white ethnicity was also a significant predictor of both maternal (Exp[*B*] 1.44; 95% CI: 1.08–1.92) and perinatal adverse outcomes (Exp[*β*} 1.36; 95% CI: 1.04–1.76). Antenatal care exclusively in the public health system was marginal but not significantly associated with increased risk of APOs (Exp[*B*] 1.58; 95% CI: 1.00–2.51; *P* = 0.051). Other sociodemographic factors, including marital status, education, income, employment, and drug use, were not significantly associated with outcomes after adjustment.

**TABLE 2:** Multivariate logistic regression of social vulnerability indicators associated with any maternal (AMO) and perinatal outcomes (APO).

Social vulnerability indicators		AMO (*n* = 1553)			APO (*n* = 1490)		
		Exp(*β*)	95% CI	*P* value	Exp(*β*)	95% CI	*P* value
Maternal age	<20 years	0.717	0.504–1.020	0.065	1.041	0.760–1.426	0.803
	>34 years	**2.478**	**1.407–4.365**	**0.002**	0.868	0.467–1.613	0.655
Marital status	Without partner	1.088	0.789–1.483	0.595	1.170	0.879–1.556	0.282
Living status	Alone	0.341	0.097–1.198	0.093	0.557	0.198–1.564	0.267
Ethnic group	Non‐white	**1.438**	**1.078–1.917**	**0.013**	**1.355**	**1.041–1.763**	**0.024**
Years of study	<12 years	0.924	0.667–1.280	0.635	0.914	0.671–1.244	0.566
Annual income (6000 BRL)	<6000 BRL	1.291	0.838–1.989	0.247	0.900	0.588–1.376	0.626
Annual income (12000 BRL)	<12 000 BRL	0.810	0.763–1.413	0.810	0.779	0.584–1.040	0.090
Labor activity	Not working	1.094	0.832–1.440	0.521	0.915	0.709–1.181	0.494
Type of antenatal care	Only public	1.249	0.780–1.999	0.354	1.581	0.997–2.505	0.051
Use of any drugs^a^	Current or during pregnancy	1.114	0.816–1.519	0.497	1.155	0.865–1.542	0.330

*Note*: Values in bold are statistically significant.

Abbreviations: AMO, adverse maternal outcomes; APO, adverse perinatal outcomes; BRL, Brazilian real; CI, confidence interval.

^a^ Missing information for 237 cases.

### 3.3 SVI testing

The multiple SVIs that were created and their correlation with AMO and perinatal outcomes are presented in Table 3. The construction of candidate SVI models was guided by the rationale of combining individual vulnerability indicators in two different ways: various logical compositions to assess how the addition and removal of each variable would affect the correlation to outcomes, and by selecting variables that presented associations in the bivariate and multivariate analyses. The process prioritized variables that either reached statistical significance or showed recurrent trends suggestive of clinical or social relevance. In particular, advanced maternal age (>34 years), non‐white ethnicity, and exclusive use of public antenatal care emerged as the most robust indicators, forming the basis of several candidate indices. Additional variables, such as annual income and employment status, were also tested in alternative combinations to explore their incremental contribution. Moreover, changes in cutoff and variations of variables, including annual income (<BRL 6000 or <BRL 12 000) and maternal age (<20 years or >34 years), were used to evaluate how they would influence the correlation to the outcomes.

**TABLE 3:** Candidate SVI models: Variable inclusion, assigned weights and selection criteria.

SVI	Maternal age		Marital status	Living status	Ethnic group	Years of study	Annual income		Labor activity	Type of AN care	Use of any drugs^c^	Index criteria
	<20 years	>34 years	Without partner	Alone	Other	<12 years	<BRL 6000	<BRL 12 000	Not working	Only public	Current or during pregnancy	
SVI_test1	0	**1**	**1**	**1**	**1**	**1**	**1**	0	**1**	**1**	**1**	All variables: >34 years + BRL 6000
SVI_test2	0	**1**	**1**	**1**	**1**	**1**	0	**1**	**1**	**1**	**1**	All variables: >34 years + BRL 12 000
SVI_test3	**1**	0	**1**	**1**	**1**	**1**	**1**	0	**1**	**1**	**1**	All variables: <20 years + BRL 6000
SVI_test4	**1**	0	**1**	**1**	**1**	**1**	0	**1**	**1**	**1**	**1**	All variables: <20 years + BRL 12 000
SVI_test5	0	**1**	0	0	**1**	0	0	0	0	**1**	0	*P* < 0.05—Chi‐square
SVI_test6	**1**	0	0	0	**1**	0	0	0	0	**1**	0	*P* < 0.05—Chi‐square with age <20 years
SVI_test7	0	**1**	**1**	0	**1**	0	**1**	0	**1**	**1**	0	*P* < 0.2—Chi‐square
SVI_test8	0	**1**	**1**	0	**1**	0	0	**1**	**1**	**1**	0	*P* < 0.2—Chi‐square with annual income < BRL 12 000
SVI_test9	0	**1**	**1**	0	**1**	0	**1**	0	0	**1**	0	*P* < 0.2—Chi‐square without labor
SVI_test10	0	**1**	0	0	**1**	0	**1**	0	0	**1**	0	*P* < 0.2—Chi‐square without labor and marital
SVI_test11	0	**1**	**1**	0	**1**	0	**1**	0	0	**1**	1	*P* < 0.2—Chi‐square plus drug use without labor
SVI_test12	**1**		**1**	0	**1**	0	**1**	0	0	**1**	1	*P* < 0.2—Chi‐square with >34 years OR <20 years use without labor
SVI_test13	0	**1.6**	0	0	**1.2**	0	0	0	0	**1.3**	0	*P* < 0.05 weighted RR max^a^—Chi‐square
SVI_test14	0	**1.25**	0	0	**1.2**	0	0	0	0	**1.3**	0	*P* < 0.05 weighted RR average^b^—Chi‐square
SVI_test15	0	**1.6**	0	0	**1.2**	0	**1.2**	0	0	**1.3**	0	SVI_test10 weighted RR max^a^—Chi‐square
SVI_test16	0	**1.25**	0	0	**1.2**	0	**1**	0	0	**1.3**	0	SVI_test10 weighted RR average^b^—Chi‐square
SVI_test17	0	**2.5**	0	0	**1.4**	0	0	0	0	**1.6**	0	*P* < 0.05 weighted Exp(*B*) max^a^—logistic regression
SVI_test18	0	**1.7**	0	0	**1.4**	0	0	0	0	**1.4**	0	*P* < 0.05 weighted Exp(*B*) average^b^—logistic regression
SVI_test19	0	**2.5**	0	0	**1.4**	0	**1.3**	0	0	**1.6**	0	SVI_test10 weighted Exp(*B*) max^a^—logistic regression
SVI_test20	0	**1.7**	0	0	**1.4**	0	**1.1**	0	0	**1.4**	0	SVI_test10 weighted Exp(*B*) average^b^—logistic regression

*Note*: Values in bold are the assigned weights for the predictors included in each model.

Abbreviations: AAO, any adverse outcome; AMO, adverse maternal outcomes; AN, antenatal; AAPO, adverse perinatal outcomes; BRL, Brazilian real; NPV, negative predictive value; PPV, positive predictive value; RR, risk ratio; SVI, social vulnerability index.

^a^ Maximum value between AMO and APO.

^b^ Average value between AMO and APO.

^c^ Missing data for 237 cases.

Across the 20 candidate indices tested (Table 4), only a subset demonstrated statistically significant associations with both maternal and perinatal adverse outcomes. Notably, models that combined advanced maternal age, non‐white ethnicity, and public‐only antenatal care—either alone or with the addition of low annual income—showed the strongest performance overall (SVI_test 5, 10 and 13–20). Among the income thresholds, a cutoff of <BRL 6000 yielded stronger discriminatory capacity than <BRL 12 000. This finding aligns with the hypothesis that only pronounced financial constraints reflect a measurable effect on maternal and neonatal health.

**TABLE 4:** SVI ROC analysis (AUROC and significance) and chi‐square testing (*P* value) for AMO and APO.

SVI	AMO			APO		
	AUROC	Sig. ROC curve	*P* value	AUROC	Sig. ROC curve	*P* value
SVI_test1	0.532	0.087	0.123^a^	0.538	**0.031**	0.291^a^
SVI_test2	0.526	0.169	0.679^a^	0.529	0.091	0.330^a^
SVI_test3	0.510	0.580	0.900^a^	0.544	**0.012**	0.318^a^
SVI_test4	0.507	0.724	1.000^a^	0.537	**0.034**	0.587^a^
SVI_test5	**0.553**	**0.004**	**0.004**	**0.546**	**0.008**	**0.018**
SVI_test6	0.512	0.512	0.065	**0.553**	**0.002**	**0.013**
SVI_test7	0.550	**0.008**	**0.043** ^a^	0.534	**0.048**	0.501^a^
SVI_test8	0.542	**0.025**	**0.017** ^a^	0.527	0.120	0.479^a^
SVI_test9	0.544	**0.018**	**0.037**	**0.549**	**0.005**	**0.023**
SVI_test10	**0.558**	**0.002**	**0.017**	0.543	**0.013**	**0.021**
SVI_test11	0.540	**0.033**	0.079^a^	**0.549**	**0.005**	**0.046** ^a^
SVI_test12	0.522	0.238	0.661^a^	**0.549**	**0.005**	0.066^a^
SVI_test13	**0.557**	**0.002**	**0.010** ^a^	**0.545**	**0.009**	**0.007** ^a^
SVI_test14	**0.553**	**0.004**	**0.010** ^a^	0.544	**0.011**	**0.007** ^a^
SVI_test15	**0.563**	**0.001**	**0.026** ^a^	0.543	**0.013**	**0.018** ^a^
SVI_test16	**0.558**	**0.002**	**0.041** ^a^	0.544	**0.012**	**0.020** ^a^
SVI_test17	**0.557**	**0.002**	**0.010** ^a^	**0.545**	**0.009**	**0.007** ^a^
SVI_test18	**0.557**	**0.002**	**0.004**	0.543	**0.014**	**0.018**
SVI_test19	**0.563**	**0.001**	**0.041** ^a^	**0.545**	**0.009**	**0.020** ^a^
SVI_test20	**0.563**	**0.001**	**0.009** ^a^	0.543	**0.013**	**0.038** ^a^

*Note*: Values in bold mean they are significant.

Abbreviations: AMO, adverse maternal outcomes; APO, adverse perinatal outcomes; AUROC, area under the receiver operating characteristic; ROC, receiver operating characteristic; SVI, social vulnerability index.

^a^ Cells with expected count <5.

The ROC analyses (Table 4) indicated modest but statistically significant predictive capacity for these models, with AUROC values ranging from 0.553 to 0.563 for maternal outcomes and 0.543 to 0.545 for perinatal outcomes. Weighted versions of the index (SVI tests 13–20), which incorporated RR or logistic regression coefficients, tended to perform slightly better than unweighted models.

When examining absolute risks (Table 5), a clear dose–response gradient emerged. In the three‐variable model (age, ethnicity, antenatal care), risk of AMOs increased from 16.4% in women without vulnerabilities to 43.8% in those with three vulnerabilities. A similar gradient was observed for perinatal outcomes, rising from 22.1% to 35.6% for up to two vulnerabilities and then to 29% for three vulnerabilities. The four‐variable model, which added low income, showed comparable risk stratification, with women presenting ≥3 vulnerabilities experiencing absolute risks above 30% for both maternal and perinatal adverse outcomes. Both models had a very similar gradient, with the four‐variable model having a slightly more linear trend when we account for the category “≥3” (applied because only five patients met the criteria for four vulnerabilities).

**TABLE 5:** AMO and APO absolute risk patterns for three‐ and four‐variable models, and the number of vulnerabilities.

Data selection	Number of vulnerabilities	AMO absolute risk	APO absolute risk
Three variables (advanced maternal age, ethnicity and type of antenatal care)	None	16.4%	22.1%
	1	21.2%	30.7%
	2	25.1%	35.6%
	3	43.8%	29.0%
Four variables (including annual income < BRL 6000)	None	16.4%	22.1%
	1	21.1%	31.1%
	2	24.5%	35.5%
	≥3	32.4%	32.6%

Abbreviations: AMO, adverse maternal outcomes; APO, adverse perinatal outcomes; BR, Brazilian real.

Figures 2 and 3 display the absolute risks for AMO and APO, respectively, according to the number of vulnerable conditions, considering up to three or four conditions.

**FIGURE 2:**
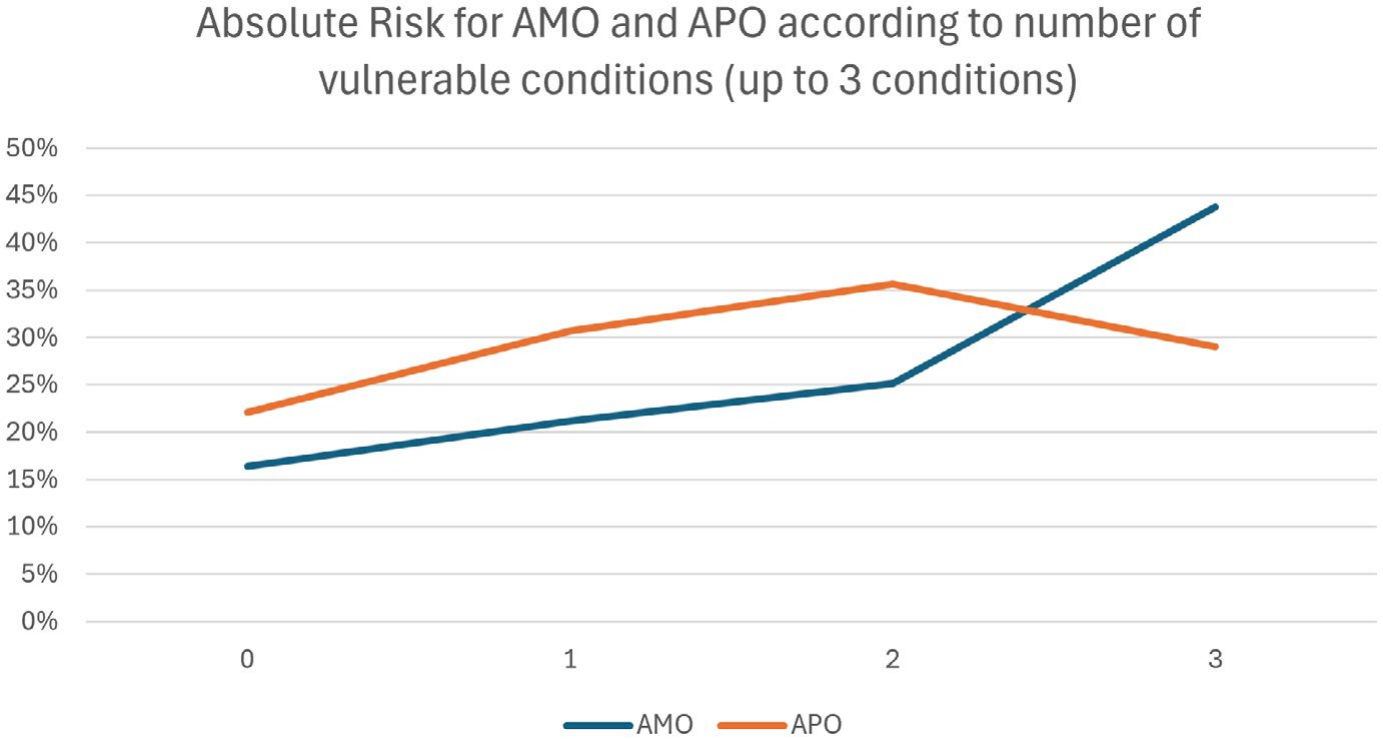
Absolute risk for adverse maternal outcomes (AMO) and adverse perinatal outcomes (APO) according to the number of vulnerable conditions (up to three conditions).

**FIGURE 3:**
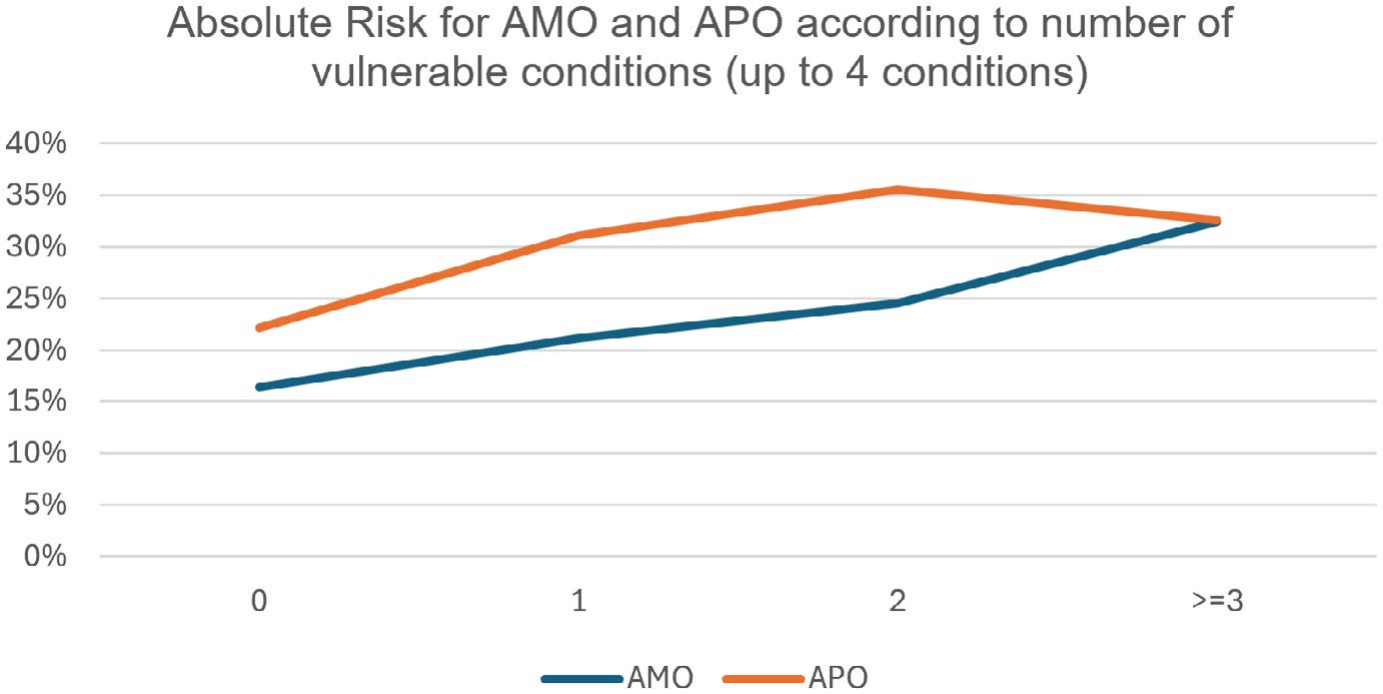
Absolute risk for adverse maternal outcomes (AMO) and adverse perinatal outcomes (APO) according to the number of vulnerable conditions (up to four conditions).

### 3.4 SVI performance

The final performance evaluation focused on the two best‐performing weighted models (SVI_test17 and SVI_test19) and is presented in Table 6. Both SVIs were created using the biggest Exp(*B*) between AMO and APO for the multivariate logistic regression model with a cutoff of ≥2.05, determined by the ROC analysis. Across maternal (AMO), perinatal (APO), and combined outcomes (AAO), sensitivity remained moderate (65%–68%) with slightly better performance in the four‐variable model, while specificity was low (around 40%), with slightly higher values in the three‐variable model. Despite these limitations, both SVIs demonstrated consistent ability to identify subgroups of women with higher SV and increased risk of adverse outcomes. Finally, Figure 4 provides a conceptual framework of this SVI for antenatal care, correlating the variables for screening with the score and its risk, besides the proposition of possible equity‐oriented interventions to try to deal with this excess social component of risk.

**TABLE 6:** SVI performance (S, E, PPV and NPV) for selected three and four‐variable models in detecting AMO, APO and AAO.

Performance	SVI_test17 (cutoff 2.05)			SVI_test19 (cutoff 2.05)		
	AMO	APO	AAO	AMO	APO	AAO
Sensitivity	67.49%	65.64%	64.71%	**68.31%**	**66.05%**	**65.31%**
Especificity	**40.86%**	**41.76%**	**42.56%**	40.02%	40.66%	41.48%
PPV	**26.03%**	**35.51%**	**47.46%**	25.99%	35.22%	47.22%
NPV	80.30%	**71.33%**	**60.07%**	**80.37%**	71.03%	59.86%

*Note*: Values in bold are the highest performance parameters for each composite outcome comparing models 17 and 19.

Abbreviations: AAO, any adverse outcome; AMO, adverse maternal outcomes; APO, adverse perinatal outcomes; NPV, negative predictive value; PPV, positive predictive value; SVI, social vulnerability index.

**FIGURE 4:**
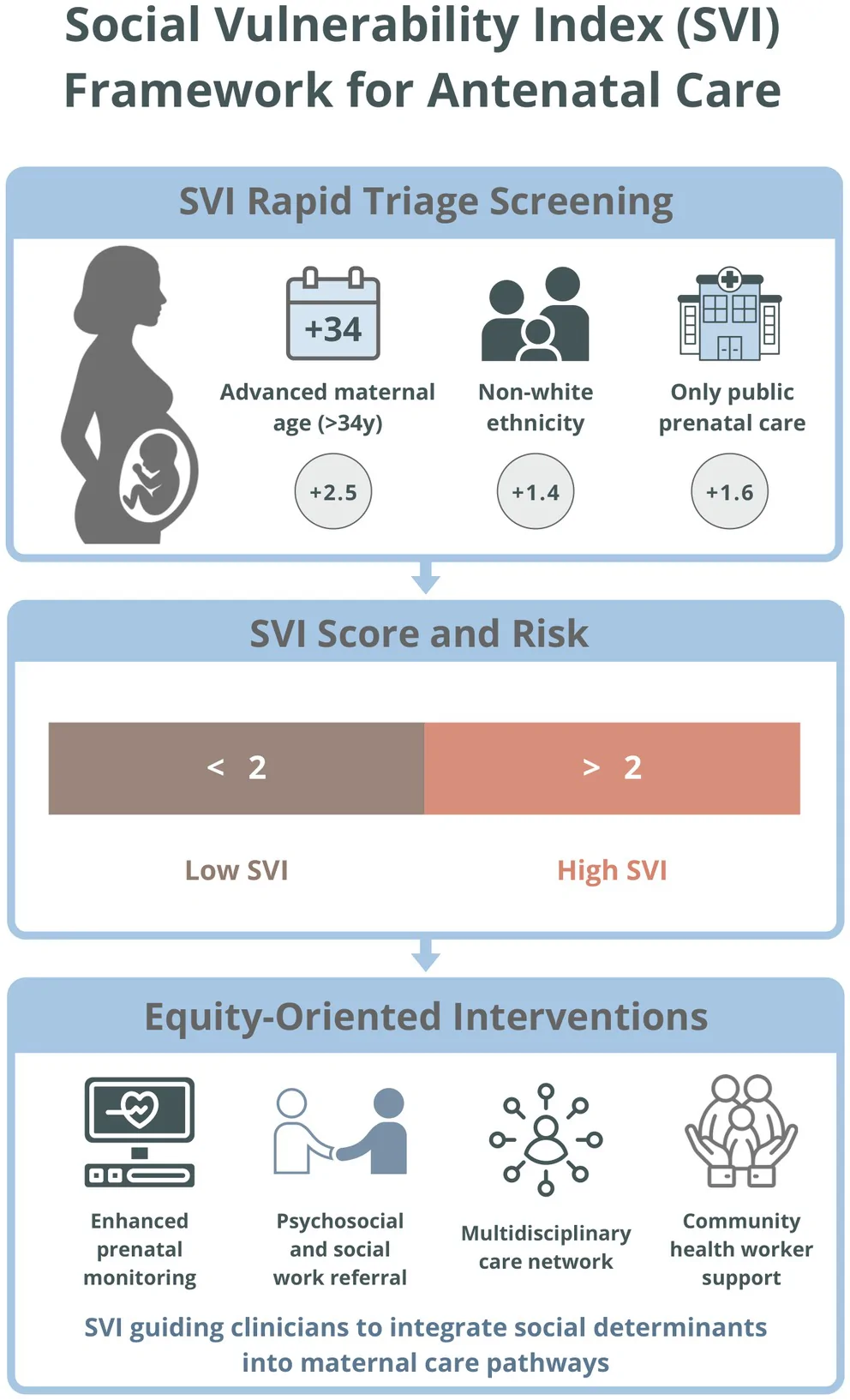
Social vulnerability index (SVI) framework for antenatal care.

## 4 DISCUSSION

The present study investigated the relationship between SV and AMO and perinatal outcomes in a population of healthy nulliparous Brazilian women. By integrating sociodemographic characteristics into a composite SVI, it was possible to demonstrate that cumulative exposure to adverse social conditions increases the likelihood of complications during pregnancy and childbirth, even among women who are otherwise healthy. The SVI combined easily obtainable individual‐level factors—advanced maternal age, non‐white ethnicity, exclusive use of publicly funded antenatal care and annual household income—and showed a clear dose–response gradient with absolute risk increasing as the number of vulnerabilities accumulated. Although the predictive performance was modest, with moderate sensitivity and low specificity, the index consistently identified subgroups at higher risk, indicating its utility as a tool for population‐level screening rather than individual diagnosis.

As the two best‐performing weighted models (SVI_test17 and SVI_test19) were very similar in predictive value, deciding the final SVI to be applied in future research and clinical practice comes down to more than just the data. It must consider applicability, reproducibility, clinical practice experience, and other criteria that should be included according to the population and settings involved, perhaps after a validation analysis including other Brazilian samples of pregnant women.

In this study, we ultimately selected the three‐variable SVI—including advanced maternal age, non‐white ethnicity, and public‐only funded antenatal care—as the preferred model. This decision was guided not only by its applicability, ease of data collection, and interpretability across diverse clinical and community contexts. Importantly, the exclusion of income from the variable selection permits easier validation in different contexts and enhances the index's reproducibility at both national and international levels, where socioeconomic thresholds may vary substantially. While its predictive performance in this healthy cohort was modest, it is plausible that in more heterogeneous populations with higher incidences of adverse outcomes, both sensitivity and specificity may differ. These results reinforce previous evidence indicating that SV contributes to disparities in maternal and neonatal outcomes through overlapping biological, socioeconomic, and structural mechanisms.^5^, ^8^, ^15^ Together, they provide empirical support for the concept that cumulative social disadvantage, even among women without major obstetric risk factors, exerts a measurable impact on pregnancy health.

Our results align with a growing international literature that both quantifies the association between SV and APOs and demonstrates that structured responses to identified vulnerability can change outcomes. Torres‐Torres et al., analyzing a large prospective Mexican cohort, found that SV at medium and high levels was independently associated with perinatal mortality, and that predictive models integrating social and clinical determinants had strong predictive value when estimating risk of perinatal death.^20^ That study highlights two important messages for the interpretation of our findings: first, social determinants can be useful predictors for severe, low‐frequency outcomes (such as perinatal death); second, maximal predictive value is obtained when social indicators are combined with clinical evaluation.

These findings support the idea that the main value of the SVI lies in identifying women who, despite being classified as clinically low risk, face multiple social disadvantages that increase their vulnerability to adverse outcomes. In this sense, the SVI enables health professionals to prioritize follow‐up, psychosocial support, and linkage to multidisciplinary services for those at higher risk. From a public health perspective, its incorporation into maternal health programs could reinforce equity‐oriented planning, directing scarce resources toward socially vulnerable women and aligning with global initiatives to reduce disparities in maternal and neonatal health. Targeted interventions may include closer antenatal monitoring, more intensive care coordination, psychosocial support and referral to multidisciplinary networks of care.

International experiences further highlight the relevance of such screening tools. In France, the PreCARE cohort showed that social deprivation was strongly associated with inadequate antenatal care utilization, despite universal coverage.^21^ This indicates that barriers extend beyond the availability of services, underscoring the need for systematic vulnerability detection to ensure appropriate follow‐up. In our context, the SVI could serve as a frontline instrument to alert primary care professionals to women at higher risk of underutilizing antenatal services, prompting proactive outreach and early interventions.

Beyond prediction, the literature strongly supports the potential for mitigation once vulnerability is detected. Crequit et al. report that a multidisciplinary personalized pregnancy follow‐up (PPFU) program for socially vulnerable women in France was associated with significant reductions in prematurity (<37 and <34 weeks) after adjustment for confounders.^22^ The intervention reduced barriers to appointment scheduling, coordinated psychosocial and addiction services, and provided dedicated navigation; concrete mechanisms that correlated to improved outcomes. This effectiveness evidence complements our proposal for the SVI: a rapid three‐item screen at first contact could operationally trigger a PPFU‐style pathway (intensified surveillance, social work, mental health and community health worker engagement), translating detection into actionable care pathways rather than passive documentation of risk. The French experience also underscores the importance of resource reallocation and local organizational commitment to multidisciplinary follow‐up to realize clinical benefits.^22^

At the same time, international implementation studies highlight the heterogeneity of vulnerability and the need to tailor responses. Mohabier et al., using the Mothers of Rotterdam cohort, used latent class analysis to identify four distinct vulnerability classes (complex, educational, social network, and financial) and showed that different clusters were associated with different durations and types of social care, implying differing support needs.^23^ This clustering evidence complements our SVI: while a short, individual‐level index is useful for rapid identification and triage, it should be complemented by mechanisms to characterize the nature of vulnerability (for example, isolation vs material deprivation vs education deficits) so that the response package is matched to the problem pattern rather than being one‐size‐fits‐all.

Qualitative and nursing literature provide crucial context on how screening and response must be delivered to be effective and equitable. Hudon and colleagues' systematic synthesis and qualitative studies emphasize that women's engagement with antenatal care is shaped not only by material barriers but also by the relational quality of care: respectful communication, cultural sensitivity, continuity, and perceived responsiveness are key determinants of follow‐up and outcome.^24^, ^25^ These studies warn against purely bureaucratic screening; identification should be followed by rapid, respectful, and useful support. Isolated screening can cause stigma or disengagement if no effective intervention is in place. Therefore, SVI implementation must be accompanied by training for providers in person‐centered communication, clear referral pathways, and safeguards against stigmatization—practical steps that echo the success factors reported for PPFU.

The strengths of this study include the use of two large, prospective multicenter cohorts with harmonized data collection, ensuring consistency across diverse regions and healthcare environments. The analytical approach combined statistical rigor and conceptual grounding, testing multiple candidate indices to identify a model that balanced feasibility with predictive performance. However, several limitations must be acknowledged. Because the study population comprised only nulliparous, healthy women who participated in research cohorts and attended multiple visits, selection bias is possible. The same could be argued, for instance, when considering the variable only publicly funded antenatal care, included in the final SVI model, based on a relatively small number of cases from private or mixed care. This could have attenuated the associations observed, as these women likely had better‐than‐average access to antenatal care. In addition, due to the baseline characteristics, the most severe adverse outcomes were also not included, such as maternal near miss and maternal death. Furthermore, an unexpected finding was the fact that the absence of a partner was not identified as a vulnerable condition, as it often appears in other studies, probably because it is also associated with the main factors selected.

Furthermore, the modest discrimination capacity indicates that the SVI should not be interpreted as a diagnostic tool for individual prognosis but rather as an individual‐level instrument for risk stratification and targeted support. Certain dimensions of vulnerability—such as domestic violence, social isolation, or community‐level deprivation—were unavailable, potentially underestimating the true burden of disadvantage. External validation in independent and more heterogeneous populations is necessary to confirm generalizability. Finally, implementation must avoid stigmatization; qualitative evidence reminds us that how screening is performed and how services respond determines uptake and benefit.

Future studies should focus on validating the SVI in diverse Brazilian and international populations, comparing its performance with neighborhood‐level deprivation indices^11^, ^13^ and assessing its predictive capacity across different risk profiles. Implementation research will be crucial to evaluate the feasibility and cost‐effectiveness of SVI‐guided interventions, as well as their impact on maternal and neonatal outcomes. Qualitative investigations exploring the experiences of women and providers could further inform the ethical and person‐centered integration of SV screening into antenatal care. Including the SVI in digital health systems, with automated scoring and referral pathways, may enhance its sustainability and scalability. Moreover, combining the SVI with clinical variables may be a pathway to develop indices with stronger predictive value and promote more specific intervention strategies.

## 5 CONCLUSION

This study demonstrates that SV, measured through a structured index, significantly influences maternal and perinatal outcomes even in populations of low clinical risk. By translating sociodemographic disadvantages into an actionable tool, the SVI provides a framework for incorporating social determinants into maternal health strategies. Its application has the potential to strengthen equity in care, enhance the allocation of resources, and contribute to reducing disparities in maternal and neonatal health.

## AUTHOR CONTRIBUTIONS

RTS, JGC and GQR: Conceived and planned the current secondary analysis from the two original cohorts. JGC, RTS, DFL, FEF, EARF, RBG, JM, MLC, RPT, DSS, KGF, MJM, JV and IMC: Developed all related procedures, implemented, and carried out the two original cohorts. GQR: Wrote the first draft manuscript under the supervision of RTS and JGC. All authors read, reviewed, and approved the final version of the manuscript.

## FUNDING INFORMATION

The first cohort, called PRETERM‐Samba, was selected from the research call “Grand Challenges Brazil: Reducing the burden of preterm birth” (no. 05/2013) jointly issued by the Brazilian National Research Council (CNPq—award 401636/2013–5) and the Bill and Melinda Gates Foundation (grant OPP1107597). The second study, called MAES, was granted by The Bill and Melinda Gates Foundation through the Grand Challenge Exploration program, call 19 (research grant OPP1182749). GQR received a scholarship from the PIBIC/CNPq from January first through August 31, 2025 (the Brazilian National Research Council) for developing the current analysis. The funders played no role whatsoever in the study design, writing of the manuscript or in the decision to submit the manuscript for publication. This article represents the views of the named authors only and does not represent the views of the sponsoring agencies.

## CONFLICT OF INTEREST STATEMENT

The authors declare that they have no competing interests.

## Data Availability

The datasets used and/or analyzed during the current study are available from the corresponding author on reasonable request. The data is already deposited in the CONMAI, the Brazilian Consortium on Maternal and Infant Nutrition, at https://hdl.handle.net/20.500.12783/168. We have permission to full access to the data used in this analysis; the primary data is also ours.
